# Targeting glycosphingolipid metabolism as a potential therapeutic approach for treating disease in female MRL/lpr lupus mice

**DOI:** 10.1371/journal.pone.0230499

**Published:** 2020-03-18

**Authors:** Tamara K. Nowling, Jessalyn Rodgers, Thirumagal Thiyagarajan, Bethany Wolf, Evelyn Bruner, Kamala Sundararaj, Ivan Molano, Gary Gilkeson

**Affiliations:** 1 Division of Rheumatology, Department of Medicine, Medical University of South Carolina, Charleston, South Carolina, United States of America; 2 Department of Public Health Sciences, Medical University of South Carolina, Charleston, South Carolina, United States of America; 3 Department of Pathology and Laboratory Medicine, Medical University of South Carolina, Charleston, South Carolina, United States of America; Instituto Nacional de Ciencias Medicas y Nutricion Salvador Zubiran, MEXICO

## Abstract

Glycosphingolipids (GSLs) hexosylceramides and lactosylceramides are elevated in lupus mice and human patients with nephritis. Whereas other renal diseases characterized by increased GSL levels are thought to be a result of upregulated GSL synthesis, our results suggest elevated hexosylceramides and lactosylceramides in lupus nephritis is a result of increased catabolism of ganglioside GM3 due to significantly increased neuraminidase (NEU) activity. Thus, we hypothesized GM3 would be decreased in lupus nephritis kidneys and blocking NEU activity would reduce GSLs and improve disease in lupus mice. Female MRL/lpr lupus mice were treated with water or the NEU inhibitor oseltamivir phosphate at the onset of proteinuria to block GSL catabolism. Age-matched (non-nephritic) female MRL/MpJ lupus mice served as controls. Renal GM3 levels were significantly higher in the nephritic MRL/lpr water-treated mice compared to non-nephritic MRL/MpJ mice, despite significantly increased renal NEU activity. Blocking GSL catabolism increased, rather than decreased, renal and urine GSL levels and disease was not significantly impacted. A pilot study treating MRL/lpr females with GlcCer synthase inhibitor Genz-667161 to block GSL synthesis resulted in a strong significant negative correlation between Genz-667161 dose and renal GSL hexosylceramide and GM3 levels. Splenomegaly was negatively correlated and serum IgG levels were marginally correlated with increasing Genz-667161 dose. These results suggest accumulation of renal GM3 may be due to dysregulation of one or more of the GSL ganglioside pathways and inhibiting GSL synthesis, but not catabolism, may be a therapeutic approach for treating lupus nephritis.

## Introduction

Systemic Lupus Erythematosus (SLE or lupus) is an autoimmune disease characterized by the development of autoantibodies, immune complex formation and deposition in target organs, and subsequent inflammation and organ damage. Glomerulonephritis, the most severe complication of lupus, affects up to 70% of lupus patients and is associated with high morbidity and mortality. Although there has been a 30% reduction in mortality of lupus nephritis (LN) patients with end-stage renal disease (ESRD) over the past 20 years [1], current therapies for treating LN are effective only in a small percentage of patients with 20–30% of patients progressing to ESRD [2–4]. New therapies are desperately needed for treating lupus patients with nephritis to prevent ESRD. The features of LN are similar to other chronic kidney diseases characterized by altered glycosphingolipid (GSL) metabolism, including diabetic nephropathy (DN) [5, 6], polycystic kidney disease (PKD) [7–9], and nephritis associated with aging [10]. Enriched in the kidney, GSLs can play a pathogenic role in progression of renal disease [6, 7, 11, 12].

We previously demonstrated GSL metabolism is altered in both lupus patients and lupus mice with nephritis. Specifically, glucosylceramides (GlcCers), lactosylceramides (LacCers), and neuraminidase (NEU) activity are elevated in the kidneys and urine of MRL/lpr and NZM2410 lupus mice with nephritis and in urine of lupus patients with nephritis compared to their non-nephritic counterparts and healthy controls [13, 14]. Unlike, DN and PKD where elevated GlcCer/LacCer appears to be due to increased GSL synthesis [5, 7, 8], our previous results suggested elevated NEU-mediated GSL catabolism may be responsible for elevated LacCer and GlcCer. NEUs remove terminal sialic acids from glycolipids, including gangliosides such as GM3, and glycoproteins. In the kidney, GSLs play an important role in the proper function of mesangial cells and podocytes [14–17]. Therefore, we hypothesized elevated LacCers/GlcCers and loss of sialic acids, specifically from gangliosides, due to increased NEU activity in the kidney, may contribute to progression of LN.

Based on our previous studies, we measured the levels of additional lipids including ganglioside GM3, the precursor for other sialic acid-containing gangliosides [18], in the kidneys of female MRL/lpr lupus mice and their non-nephritic counterparts (MRL/MpJ). We also tested if blocking NEU activity in MRL/lpr female mice would correct altered GSL metabolism and improve disease. Nephritic MRL/lpr mice showed elevated levels of renal GM3 compared to age-matched MRL/MpJ mice and MRL/lpr mice treated with the NEU inhibitor resulted in a further trend of increased GM3 levels. Unexpectedly, NEU inhibitor treatment slightly increased GSLs and failed to significantly impact renal or immune disease measures in the MRL/lpr mice. We then performed a pilot treatment study in MRL/lpr mice with an inhibitor of GlcCer synthase, which resulted in a significant negative correlation between inhibitor dose with renal GSLs levels and splenomegaly. There was also a trend towards lower serum IgG levels at the higher doses of the inhibitor. Together these results demonstrate GM3 accumulation in MRL/lpr nephritic mice despite increased elevated renal NEU activity, and blocking the synthesis pathway may normalize renal GSL metabolism and reduce disease measures.

## Materials and methods

### Ethics statement and mouse strains

All animal studies and methods of euthanasia were approved by the local Institutional Animal Care and Use Committee (IACUC). The oseltamivir phosphate study was also approved by the Department of Defense IACUC. Breeding pairs of MRL/lpr mice were purchased (Stock #000485, Jackson Laboratory, Bar Harbor, ME) and bred in-house to obtain female littermates for all studies. Breeding pairs of MRL/MpJ mice were purchased (Stock #000486, Jackson Laboratory, Bar Harbor, ME) and bred in-house to obtain female littermates. All mice used in the presented studies were female. Mice were maintained on a 12 h light-dark cycle in groups of 3–5 per cage in a pathogen free environment with access to food and water ad libitum throughout the study. Once a week, mice were housed individually in metabolic cages to collect urine during the study as described below after which time they were returned to their same group housing. Mice exhibiting respiratory distress due to gavage, or signs of advanced disease including skin rash over greater than 10% of their body surface, >15% loss of body weight, or decreased grooming prior to study endpoints were euthanized.

### Oseltamivir phosphate treatment study

Beginning at 8 weeks of age, 30 MRL/lpr female mice were monitored bi-weekly for onset of proteinuria using Chemstrip 7 protein strips (Roche, Indianapolis, IN). When at least half of the mice showed measurable proteinuria (12–13 weeks of age), mice were divided into oseltamivir phosphate (OP)- and water-treatment groups with equivalent numbers of mice with high and low levels of proteinuria in each group (based on protein strip estimates). Mice administered OP at 10 mg/kg/day have similar plasma levels of oseltamivir carboxylate, the active metabolite of OP, with the same area under the curve (AUC) as humans administered OP at 75 mg/kg twice daily [19]. Moreover, a 10 mg/kg dose in mice was effective in blocking mammalian neuraminidase activity *in vivo* [20, 21]. Therefore, mice received vehicle (water) or 10 mg/kg/day oseltamivir phosphate (OP) (Santa Cruz Biotechnology sc-208135A; Dallas, TX) by gavage daily for four weeks. The study was initiated with 16 mice in the water treatment group and 15 mice in the OP treatment group. One mouse in the water treatment group was euthanized during the first week of treatment due to complications from gavage and removed from the study. Hence, there were 15 MRL/lpr mice in each treatment group. One mouse from each group died and one mouse from each group was euthanized prior to the end of the study. All mice that died or were euthanized had urine albumin >50 mg/day for at least a week and enlarged lymph nodes. Renal samples from the euthanized mice were included in the endpoint analyses and results showed pathological changes consistent with renal disease for both mice. Thus, we concluded the four study mice succumbed or were succumbing due to advanced disease. Gavage was performed at the same time of day for all mice throughout the four-week treatment period. Treatments were performed in two successive sets of mice (6–8 mice per treatment group) due to constraints on metabolic cage availability and personnel for performing gavage. Ten female MRL/MpJ littermates were used as age-matched controls and remained untreated. Twenty-four-hour (24 h) urine and serum were collected one day prior to beginning treatment and then weekly throughout the study. Body weight was monitored throughout the study. Mice were euthanized and organs immediately harvested for analyses. One kidney was flash frozen and one was fixed and paraffin embedded. Approximately one-fourth of the spleen was used for flow cytometry and the rest was flash frozen and stored.

### Genz-667161 treatment study

Fifteen female MRL/lpr littermates were monitored bi-weekly beginning at 8 weeks of age for onset of proteinuria using Chemstrip 7 protein strips (Roche, Indianapolis, IN). When at least half of the mice showed measurable proteinuria (12–13 weeks of age), the mice were randomly assigned to a treatment with three mice per group and their food switched to the same chow containing Genz-667161, a glucosylceramide synthase inhibitor developed by Sanofi Genzyme (Waltham, MA). The drug was formulated into the chow by Harlan Laboratories (Madison, WI) such that the mice received approximately 0, 1, 3, 10, or 20 mg/kg/day for four weeks. Twenty-four-hour (24 h) urine and serum were collected one day prior to beginning treatment and then weekly throughout the study. Body weight was monitored throughout the study. At completion of the study, mice were euthanized and organs removed. The spleen was weighed and flash frozen, one kidney was flash frozen, and one kidney was fixed and paraffin embedded.

### Neuraminidase activity assay and lipid measures in kidney homogenates, urine, or mesangial cells

Frozen kidney cortex tissue was homogenized in 20 mM Tris HCl pH 7.4 buffer containing protease inhibitors (Roche, Indianapolis, IN) as described previously [14]. Protein concentration was measured using the BCA assay. Renal neuraminidase activity of kidney homogenates (250 μg) was measured using Amplex Red Neuraminidase Assay Kit according to manufacturers’ protocol (Invitrogen A22178; Carlsbad, CA). Mesangial cell neuraminidase activity was measured in live cells as follows. Cells were washed with 37°C warmed sterile filtered Tris-buffered saline (TBS) pH 7.4 and incubated with 15 μM substrate 2′ (4-methylumbelliferyl)-α-D-*N*-acetylneuraminic acid (4MU-NANA) in 37°C warmed sterile filtered TBS (Sigma Aldrich; St. Louis, MO). Fluorescence intensity (excitation: 365 nm, emission: 460 nm) was measured on a SpectraMax i3c fluorometer with SoftMax Pro 6 software (Molecular Devices, San Hose, CA) at baseline prior to substrate addition and in the presence of substrate. Relative fluorescence units (RFU) per 2x10^4^ cells after 45 min incubation with substrate are presented on the graph. Total protein content was consistent between untreated and treated cells. Ceramides (Cers), hexosylceramides (HexCers), lactosylceramides (LacCers), sphingomyelins (SMs), Sphingosine-1 Phosphate (S1P), Sphingosine (So), Dihydrosphingosine-1 Phosphate (DHS1P), or Dihydrosphingosine (DHSo) were quantified in kidney homogenates (1 mg), 24-hr urine collections (100 μl), or mesangial cells (1x10^6^ cells) by the Virginia Commonwealth University (VCU) Lipidomics/Metabolomics Core Facility or Medical University of South Carolina Lipidomics Core Facility, and Ganglioside GM3s were quantified by the VCU Lipidomics Facility as described previously [13, 22]. Lipid levels in urine were normalized to urine creatinine. Since the OP study was performed in two sets, relative levels for each GSL were calculated within each set of mice, sets were combined, and presented as fold change.

### Urine albumin and creatinine ELISA

Urine was collected over a 24 h period using individual metabolic cages once a week. Urine was collected into tubes containing an antibiotic cocktail of Ampicillin, Gentamicin, and Chloramphenicol to prevent bacterial growth. Total albumin levels were measured by ELISA as previously described [13]. Briefly, wells were pre-coated overnight at 4°C with goat anti-mouse Albumin (Fitzgerald Industries, Acton, MA; Cat# 70R-AG001) in 0.05M Carbonate Bicarbonate, blocked with PBS-Tween20 (PBST) + 0.25% gelatin (PBST-GT) and washed with (PBST). Mouse albumin standard (Sigma-Aldrich, St. Louis, MO; cat# A-3559) and urine were serially diluted with PBST-GT directly in the plate and incubated. After washing with PBST, albumin was detected with HRP-conjugated goat-anti-mouse Albumin (Fitzgerald Industries, Acton, MA; Cat# 60R-AG002) and 3,3’,5,5’–tetramethylbenzidine (TMB, Sigma, St. Louis, MO) in citrate buffer (3% H_2_O_2_ + 0.1M, pH 4.0). The reaction was quenched with 1M phosphoric acid stop solution and OD measurements read at 450nm. OD values were calculated using values that fell within the linear portion of the standard curve and averaged for each sample. Urine creatinine was measured using the Jaffe assay [23]. Briefly, creatinine standard (Sigma-Aldrich, St. Louis, MO) and urine sample were incubated for 10 min at room temperature with distilled water, 0.75M NaOH and 1% picric acid and optical density read at 492nm.

### Serum IgG ELISA

Total IgG levels in mouse sera were measured by ELISA as described previously [24]. Briefly, wells were pre-coated overnight with goat anti-mouse IgG (Southern Biotech, Birmingham, AL; cat#1030–01) in 0.05M Carbonate Bicarbonate buffer. Blocking and sample dilutions were performed with 1% BSA in PBS-Tween20 (BSA/PBST). Mouse IgG (Sigma-Aldrich cat# I-8765; St. Louis, MO) served as the standard and diluted in BSA/PBST. IgG was detected with HRP-conjugated goat-anti-mouse IgG (Southern Biotech, Birmingham, AL; cat# 1030–05) diluted in BSA/PBST. Wells were then incubated with TMB, reaction was quenched with 1M phosphoric acid stop solution, and OD measurements read at 450nm. OD values were calculated using values that fell within the linear portion of the standard curve and averaged for each sample.

### Flow cytometry

Approximately one-quarter of each spleen was dissociated in RPMI 1640 as described previously [24] to generate single-cell suspensions. Red blood cells were lysed in ACK lysis buffer (Lonza, Walkersville, MD). Cells were suspended in flow buffer (PBS, 1% fetal bovine serum, 0.1% sodium azide) and Fc receptors blocked with anti-mouse CD16/CD32 (BD Bioscience, San Jose, CA). Live-dead fixable aqua dead cell stain (Thermo-Fisher, Waltham, MA) was used according to manufacturer’s instructions to gate on live cells. The percentage of T cells expressing markers of activation were measured using a BD LSR Fortessa flow cytometer after staining with anti-CD3-FITC, CD44-APC, and CD62L-PE antibodies (clones: 145-2c11, IM7, and MEL-14 respectably, BD Biosciences, San Jose, CA). Live cells were gated on CD3 and then analyzed for CD44 and CD62L staining.

### Renal pathology assessment

Fixed kidney sections from each of the study mice were stained with H&E or PAS and evaluated by a pathologist blinded to the treatment. Renal pathology scores were based on glomerular inflammation, proliferation, crescent formation, necrosis, and interstitial inflammation on a scale of 0 to 3 for each feature. Scores for each feature were totaled and presented in figures. Any observed crescent formation and necrosis were given double scores due to severity of those lesions.

### Culture and stimulation of mesangial cells

Primary mesangial cells (MCs) were generated from glomeruli isolated from six week-old MRL/lpr female mice, characterized/phenotyped, and cultured as described previously [14]. MCs were stimulated with 10% serum collected and pooled from 16–18 week-old MRL/lpr mice (referred to as lupus serum). For OP treatments, MCs were incubated with 500 μM OP 16–18 hrs prior to addition of lupus serum for 3 hrs. Significant production of IL-6 after addition of lupus serum was used to confirm stimulation in each experiment as demonstrated previously [14]. NEU activity and lipids were measured as described above.

### Statistical analyses

All analyses were conducted in SAS v. 9.4 (SAS Institute, Cary, NC). In the MRL/lpr OP treatment study, associations between animal type (MpJ, lpr OP-treated, and lpr water-treated) with fold change in renal GM3, SMs, sphingolipids, Ceramide, HexCers, and LacCers for chain lengths C16, C22, C24, C24_1 and Total of all chain lengths (C14-C26) were evaluated using an ANOVA approach. Pairwise differences between animal types were evaluated using linear contrasts with a Bonferroni adjustment. The associations between animal type with renal score, overall change in urine albumin, spleen weight, spleen:body weight ratio, and activated T cells were examined using an ANOVA or Kruskal-Wallace approach where appropriate. The association between animal type with urine albumin and NEU activity over time were evaluated using a linear mixed model (LMM) approach. LMMs included fixed effects for animal type, time, and the interaction between type and time and a random mouse/sample effect to account for correlation between repeated measures in the same mouse/sample over time. Pairwise differences between animal types were evaluated using linear contrasts with a Bonferroni adjustment.

In the MRL/lpr Genz-667161 treatment study, dose of Genz-667161 was considered both as continuous, to determine the potential dose response relationship, and categorical to examine mean response in each dose group. The association between continuous dose with lipid profiles and with spleen weight outcomes was evaluated using Pearson’s or Spearman’s rank correlation as appropriate. The associations between treatment dose with lipid profile and spleen weight outcomes were examined using an ANOVA approach. Pairwise differences between treatment groups were evaluated using linear contrasts. No adjustment for multiple comparisons was made due to the exploratory nature of this experiment.

For the MC culture experiment, association between treatment of lupus serum in the absence or presence of OP with GSL levels were evaluated using a linear mixed model approach. Models with one treatment variable included a fixed effect for treatment. All experiments were performed twice with similar results, data were combined for the statistical analyses, and representative graphs are presented. Model assumptions were checked graphically and transformations were considered as needed. Pairwise differences between groups were evaluated using linear contrasts and a Bonferroni correction was applied to adjust for multiple comparisons. Adjusted p-values are presented on the graphs.

## Results

### Effects of NEU inhibition on renal disease in MRL/lpr mice

**Fig 1:**
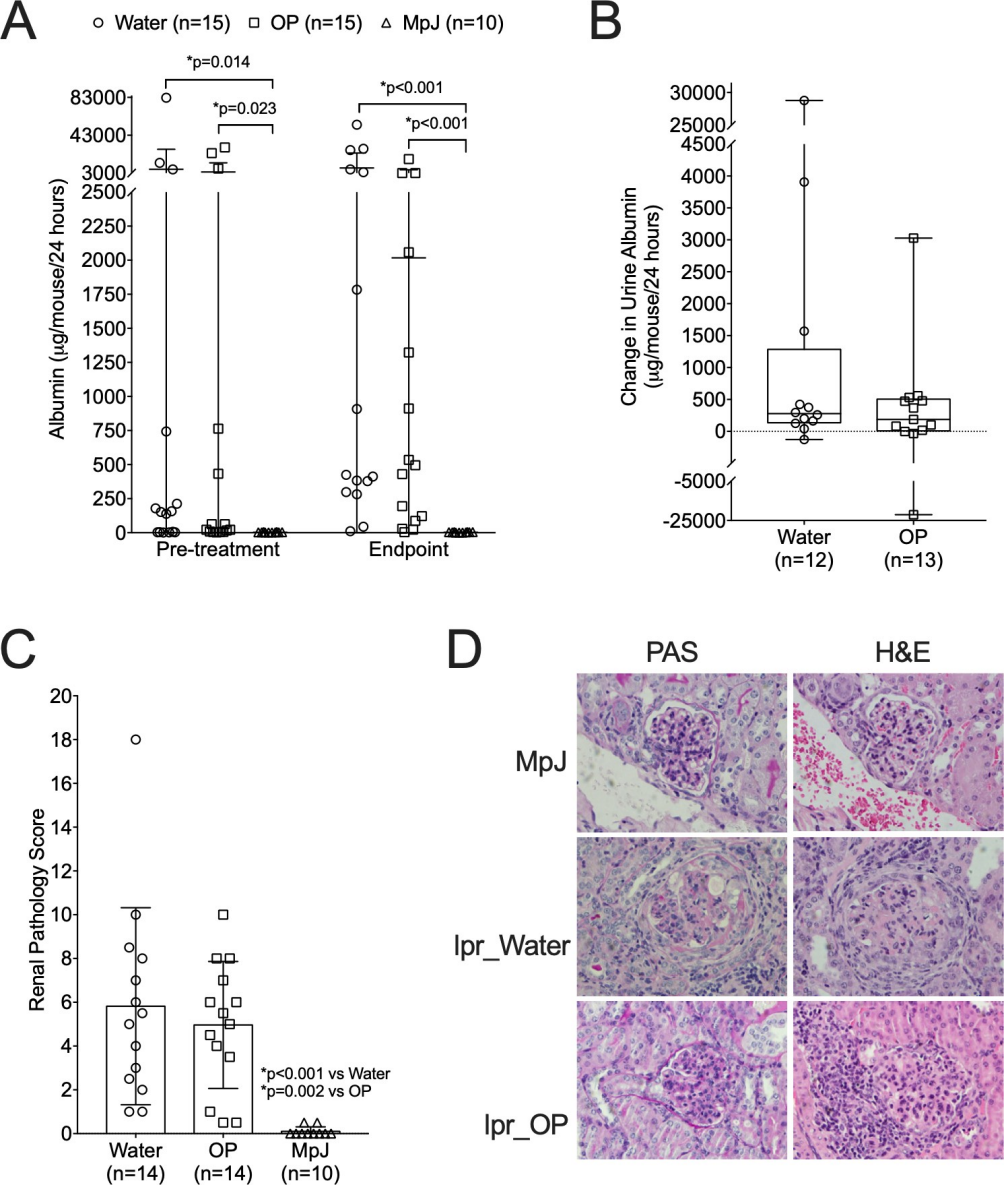
Renal disease measures are not significantly impacted by OP treatment in MRL/lpr mice. MRL/lpr female mice were treated with vehicle (Water) or oseltamivir phosphate (OP) at 12–13 weeks of age for 4 weeks. Untreated age-matched MRL/MpJ (MpJ) female mice served as non-nephritic lupus controls. Urine albumin was measured in 24 hr urine samples one day prior to beginning treatment (Pre-treatment) and one day prior to euthanasia (Endpoint). Both the absolute levels (A) and the change from Pre-treatment to Endpoint (B) in urine albumin are presented. (C) Renal pathology scores at the endpoint of the study. Means with standard deviations are presented. (D) Renal sections stained with H&E or PAS from the MRL/MpJ (MpJ) and MRL/lpr water-treated (lpr_Water) and OP-treated (lpr_OP) mice.

To test if pharmacologically reducing neuraminidase (NEU) activity, which is elevated in MRL/lpr mice [13], would normalize GSL metabolism and improve disease, female MRL/lpr mice received 10 mg/kg of the NEU inhibitor oseltamivir phosphate (OP) or water daily for four weeks. Ten age-matched untreated MRL/MpJ mice were used as non-nephritic controls. Effects of OP treatment on kidney disease were analyzed by measuring proteinuria and assessing renal pathology. Albuminuria levels on average were similar between the water- and OP-treated MRL/lpr (lpr) mice prior to beginning treatment. Both lpr groups had significantly higher albuminuria than age-matched MRL/MpJ (MpJ) controls prior to beginning treatment and at the endpoint (Fig 1A). While most mice in both of the lpr groups had increases and a few mice had decreases in proteinuria over the treatment period (Fig 1A), on average, there was not a significant difference in the change in urine albumin between the OP- and water-treated groups (Fig 1B). There also was no significant difference in total renal pathology scores between the water- and OP-treated lpr groups, and both lpr groups had significantly higher renal pathology scores than the MpJ group (Fig 1C). Examples of pathological features observed in both the lpr water-treated (crescents) and OP-treated (hypercellularity) mice are shown compared to normal pathology in the MRL/MpJ mice (Fig 1D).

### Effects of NEU inhibition on systemic disease in MRL/lpr mice

**Fig 2:**
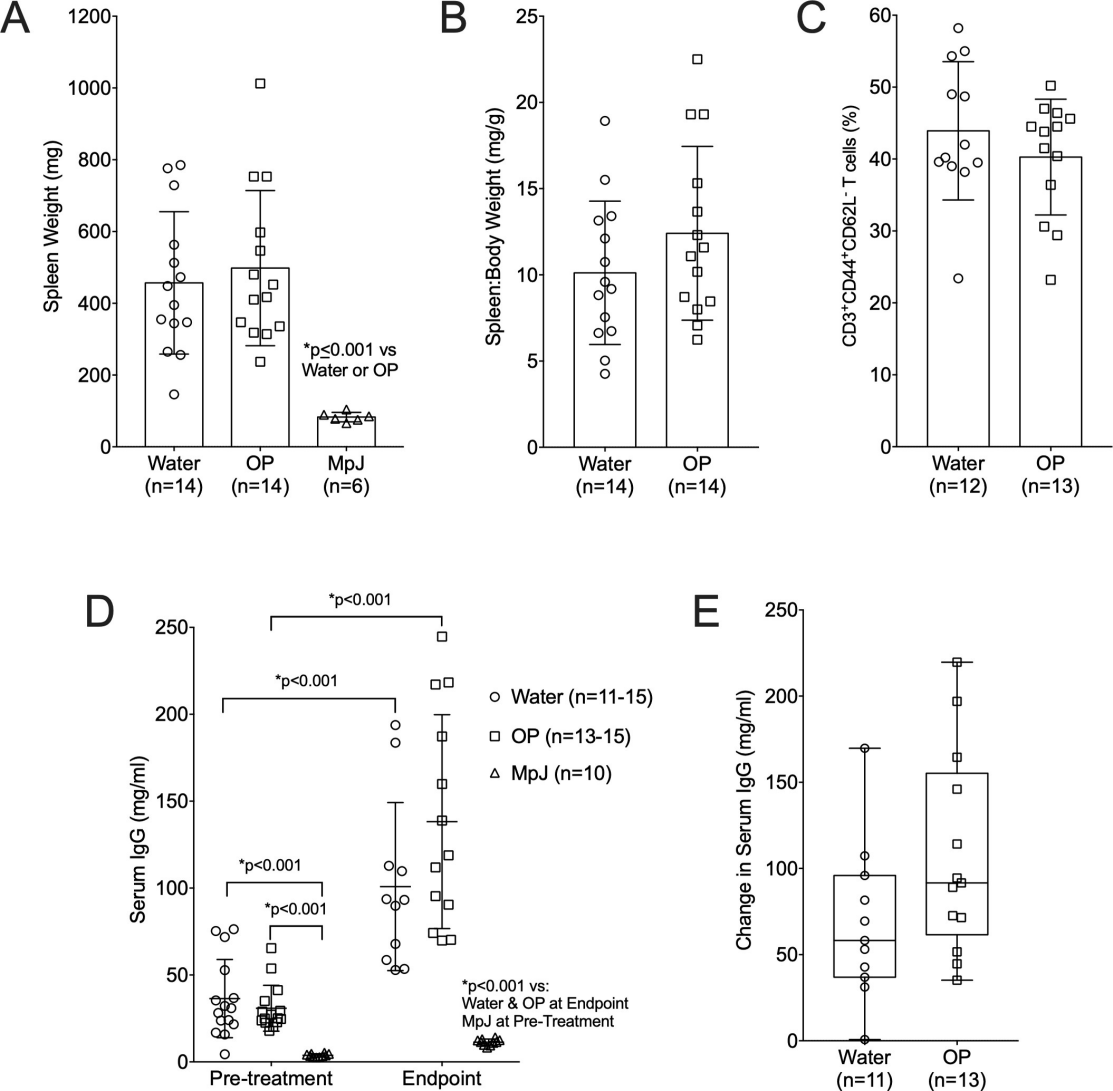
Systemic disease measures are not significantly impacted by OP treatment in MRL/lpr mice. Splenomegaly in the same mice as in Fig 1 was determined by absolute spleen weight (A) and as spleen to body weight ratio (B). (C) Percentages of activated/memory T cells (CD3^+^ CD44^+^ CD62L^-^) were measured in spleen by flow cytometry. IgG levels were measured by ELISA in serum samples collected one day prior to treatment (Pre-treatment) and one day prior to euthanasia (Endpoint) with absolute levels (D) and change in levels from Pre-treatment to Endpoint (E) presented. Means with standard deviations are presented. Water, water-treated MRL/lpr mice; OP, OP-treated MRL/lpr mice; MpJ, MRL/MpJ (untreated) mice.

We previously demonstrated Neu1 levels and LacCers levels are elevated in MRL/lpr T cells from nephritic mice compared to age-matched non-nephritic mice and parallels their hyperactivation *ex vivo* [24]. Therefore, we investigated if OP treatment impacted splenomegaly, the percentage of activated T cells, and serum IgG levels. As expected, the water-treated lpr mice had significantly larger spleens than the MpJ mice (Fig 2A). However, there were no differences in spleen size (Fig 2A) or spleen to body weight ratio (Fig 2B) and no differences in the percentage of activated T cells (Fig 2C) between the OP-treated and water-treated MRL/lpr groups. Serum IgG levels were significantly higher in the water-treated lpr mice compared to the MpJ mice just prior to treatment and at the study endpoint and both groups showed significant increases over the four weeks of the study (Fig 2D). Although not significant, there was an unexpected slight increase in the absolute levels of serum IgG at the endpoint (Fig 2D) and in the change in levels from pre-treatment to endpoint (Fig 2E) in the OP-treated compared to the water-treated lpr groups.

### Effects of NEU inhibition on renal GSL metabolism in MRL/lpr mice

**Fig 3:**
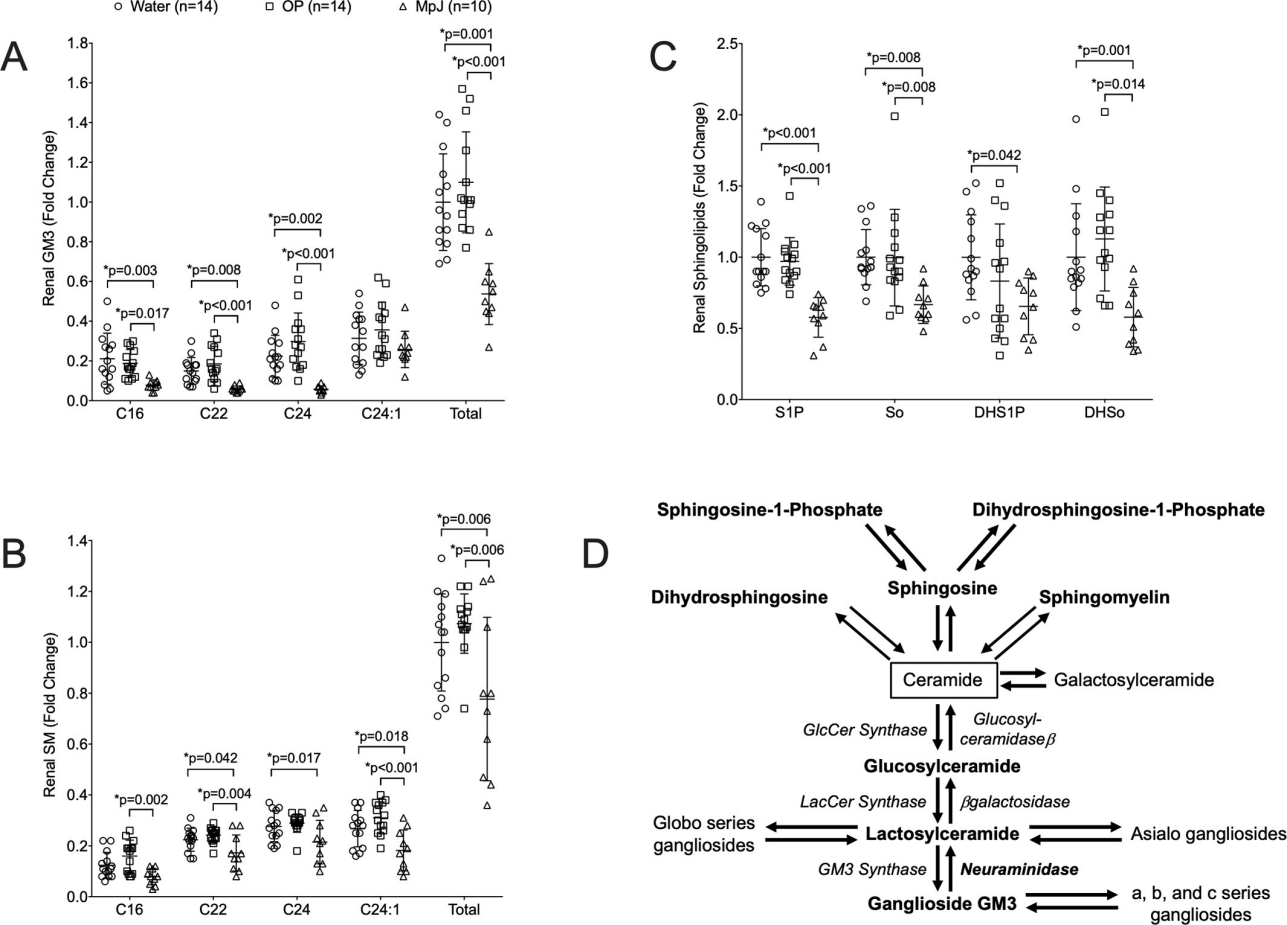
Elevated renal GM3s and Sphingolipids in nephritic MRL/lpr are not significantly impacted by treatment with OP. Renal lipids were quantified in renal cortex homogenates from the same mice as in Fig 1 at the endpoint of the study. Major chain length species (C16, C22, C24, C24:1) and total of all chain lengths (Total) are presented for each animal for: (A) Ganglioside GM3s and (B) Sphingomyelins (SMs). Overall levels are presented for each animal for: (C) Sphingosine-1 phosphate (S1P), sphingosine (So), dihydrosphingosine-1 phosphate (DHS1P), and dihydrosphingosine (DHSo). Means with standard deviations are provided on all graphs. Water, water-treated MRL/lpr mice; OP, OP-treated MRL/lpr mice; MpJ, MRL/MpJ (untreated) mice. (D) Schematic of GSL metabolism and the pathways feeding into it. Enzymes are in italics. Lipids and enzymes in bold are elevated in kidneys of nephritic female MRL/lpr compared to age-matched female MRL/MpJ mice.

We previously demonstrated significantly elevated levels of renal LacCers, GlcCers, and NEU activity in nephritic MRL/lpr (lpr) mice compared to age-matched non-nephritic MRL/MpJ (MpJ) lupus mice and healthy C57BL/6 controls, but the levels of ceramide and the message levels of synthesis pathway enzymes were not significantly elevated [13]. These results suggested NEU-mediated catabolism of gangliosides may be responsible for the elevated LacCer or GlcCer levels in the kidney. Therefore, we compared the levels of renal ganglioside GM3, the simplest sialic acid-containing ganglioside (LacCer with a single sialic acid) and most abundant ganglioside [25], in the water-treated lpr and untreated MpJ mice at the endpoint. Surprisingly, all the major GM3 chain-length species except C24:1, as well as total (all chain lengths from C14 to C26:1) GM3 levels, were significantly elevated in the lpr compared to the MpJ mice (Fig 3A). We also measured the levels of lipids that feed into the GSL pathway through ceramide, including sphingomyelin (SM), spingolipid-1 phosphate (S1P), sphingosine (So), dihydrosphingolipid-1 phosphate (DHS1P), and dihydrosphingosine (DHSo). Excluding C16, all of the SM chain lengths and total SM levels are significantly elevated (Fig 3B) as are S1p, So, DHS1P, DHSo (Fig 3C) in the kidney of the water-treated lpr mice compared to their age-matched non-nephritic MpJ counterparts. Fig 3D depicts the lipids and enzymes in the GSL pathway and the lipids that feed into the GSL pathway through Cer. Enzymes and lipids in bold text were demonstrated to be elevated in the kidneys of female nephritic lpr compared to female MpJ age-matched (non-nephritic) mice previously [13] and in this study.

Inhibiting NEU activity and blocking the catabolism of GM3 to LacCer was expected to result in accumulation of renal GM3 levels in the OP-treated mice. Although differences were not significant, the longer chain-length GM3 species (C24, C24:1) and total GM3 levels showed a trend towards being increased in the kidneys of OP-treated mice compared to the water-treated lpr mice. This was supported by the smaller p values for OP-treated lpr versus MpJ compared to those for the water-treated lpr versus MpJ for C22, C24, and total GM3. The OP-treated lpr mice also showed a trend towards slight increases in renal SM (Fig 3B), but not for S1P, So, DHS1P, and DHSo levels (Fig 3C) compared to water-treated lpr mice. The elevated renal levels of SMs, S1P, So, DHS1P, and DHSo in MRL/lpr mice with nephritis compared to the non-nephritic lupus MRL/MpJ mice suggest GSLs may be elevated due in part to increased synthesis through ceramide (Fig 3D).

**Fig 4:**
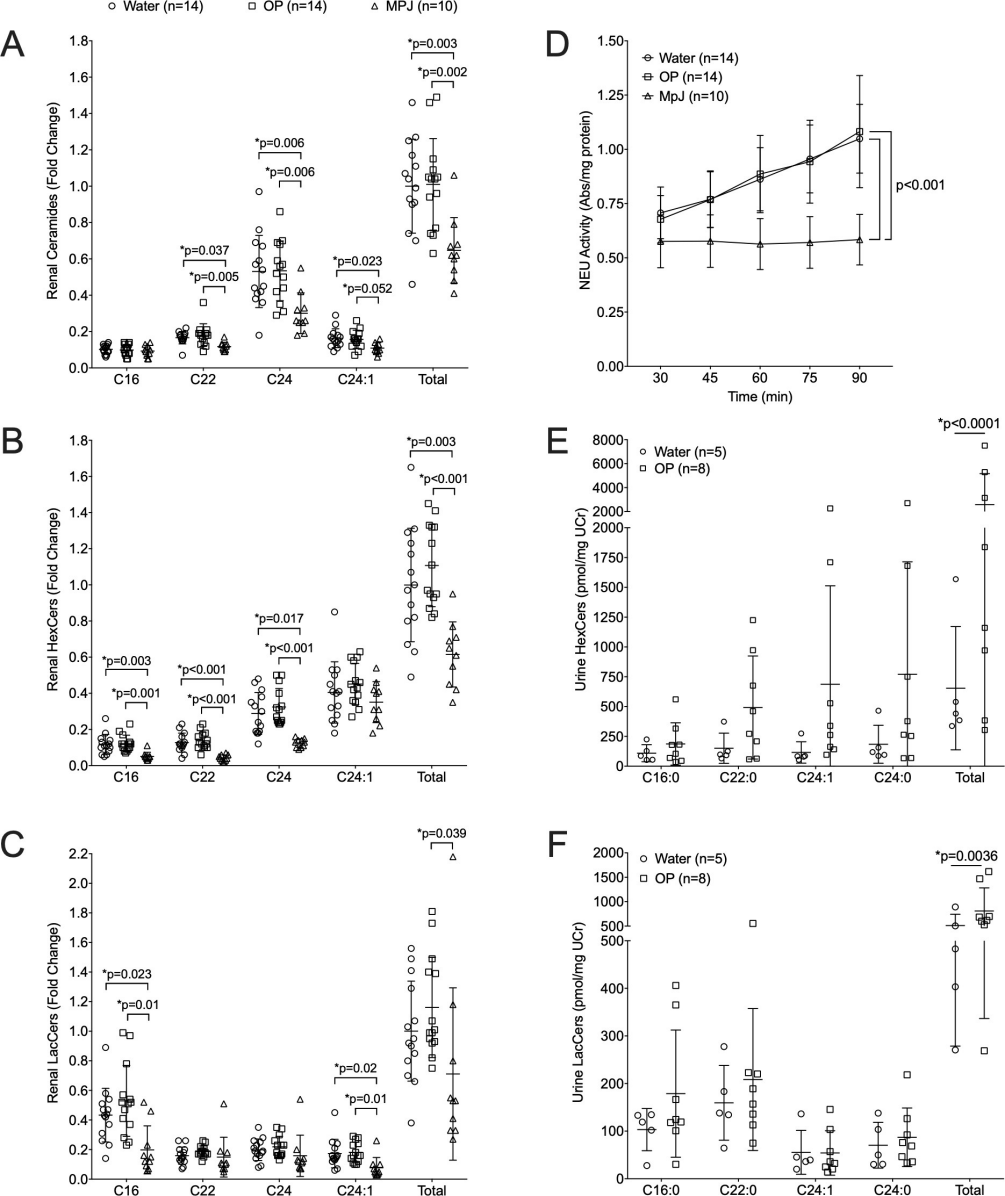
Renal and urine HexCers and LacCers are increased in OP-treated MRL/lpr mice. Renal GSLs were quantified in renal cortex homogenates from the same mice as in Fig 1 at the endpoint of the study. (A) Ceramides (Cers). (B) Hexosylceramides (HexCers). (C) Lactosylceramides (LacCers). Major chain length species (C16, C22, C24, C24:1) and total of all chain-lengths (Total) of each lipid presented for each animal are presented in the graphs. (D) Neuraminidase (NEU) activity was quantified in kidney cortex homogenates. NEU activity (absorbance, Abs) was quantified within each homogenate at 15 min intervals, normalized to total protein, and presented as average absorbance per mg protein for each time point. HexCers (E) and LacCers (F) were measured in 24-hr urine collections at the endpoint of the study and normalized to urine creatinine (UCr). Means with standard deviations are provided for all graphs. Water, water-treated MRL/lpr mice; OP, OP-treated MRL/lpr mice; MpJ, MRL/MpJ (untreated) mice.

Previously, we observed most of the renal hexosylceramides (HexCers) chain lengths, composed of GlcCers and galactosylceramides (GalCers), were significantly elevated in lpr compared to MpJ female mice [13]. We further demonstrated the increased levels of HexCers were due to increased GlcCers. Therefore, due to ease and cost, we measured HexCer levels and consider them representative of GlcCers. Similar to our previous results, we observed a significant increase in renal C24 Cer in the water-treated lpr compared to the MpJ mice (Fig 4A). However, we observed renal C22, C24:1, and total Cers were also significantly elevated in the water-treated lpr compared to the MpJ mice. Elevated levels of C16, C22, C24, and total HexCers (Fig 4B) and C16, C24:1, and total LacCers (Fig 4C) were all elevated in the water-treated lpr compared to the MpJ mice as observed previously [13]. The overall levels of renal HexCers and LacCers were expected to decrease in the OP-treated mice due to decreased NEU-mediated catabolism of gangliosides to LacCer (and subsequent catabolism to GlcCer). However, Cers levels were not significantly different (Fig 4A) and there was a trend towards increased levels of renal HexCers (Fig 4B) and LacCers (Fig 4C) in the OP-treated compared to the water-treated lpr nice.

To determine if the OP treatment impacted renal NEU activity as expected, we measured renal NEU activity. Both the water-treated and OP-treated lpr mice had a significant increase in NEU activity compared to MpJ mice (Fig 4D, p<0.001) as expected based on previous observations [13]. However, NEU activity levels were not significantly different between the water- and OP-treated lpr mice. GSL levels and NEU activity in the kidney could only be measured at the endpoint of the study. Since the kidneys were collected 24 hrs after the last dose of OP, 12–14 hrs beyond the half-life of the drug and NEU activity may have recovered by the time of organ collection, we measured lipid levels in the urine samples of the first set of MRL/lpr water- and OP-treated mice. Urine was collected over 24 hrs and may be a better indicator if OP impacted GSL metabolism as it would capture the period during highest drug activity. Urine levels of most HexCers and LacCers in the endpoint urine samples were higher in the OP-treated mice compared to the water-treated mice, and significantly higher when totaling all chain lengths; HexCers (Fig 4E, p<0.0001) and LacCers (Fig 4F, p = 0.0036). Similar increases were observed in the Cers (S1A Fig) and SMs (S1B Fig) in the endpoint urine samples from OP-treated mice with significant increases observed in total Cers (p = 0.0006), and in C16 (p = 0.0012) and total (p<0.0001) SMs compared to water-treated mice.

**Fig 5:**
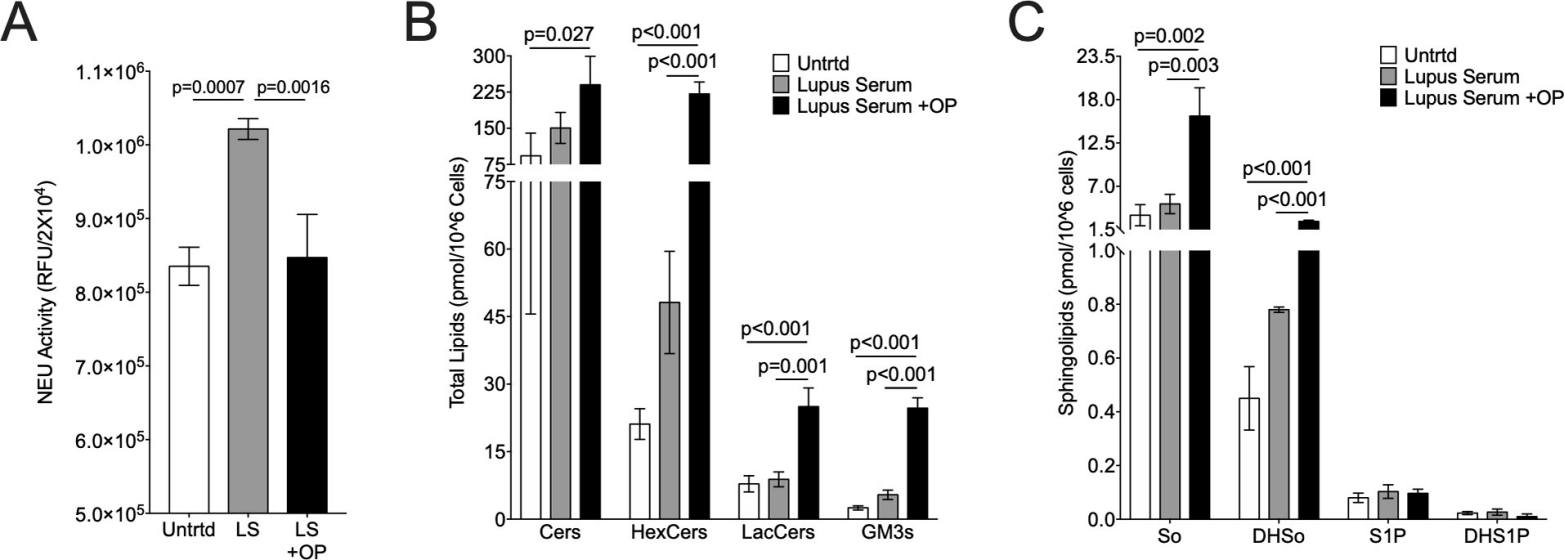
GSLs are significantly increased in OP-treated primary mesangial cells. Primary MRL/lpr mesangial cells were incubated with OP or vehicle (water) prior to addition of 10% MRL/lpr lupus serum or left untreated. A) NEU activity was measured in live cells. B) Levels of Cers and glycosphingolipids HexCers, LacCers, and GM3s were quantified for each chain length and totaled. C) Sphingolipids sphingosine (So), dihydrosphingosine (DHSo), spingosine-1 phosphate (S1P), and dihydrospingosine-1 phosphate (DHS1P) were quantified. Lipid levels are presented as pmol lipid per 10^6^ cells. Means with standard deviations are provided for all graphs. P-values were calculated as described in the materials and methods.

To confirm the observed increases in urine lipids at the endpoint were not due to differences in the starting baseline (pre-treatment) levels, the change in urine lipids from baseline to endpoint samples were calculated. These results confirm urine total HexCers (S1C Fig), LacCers (S1D Fig), Cers (S1E Fig), and SMs (S1F Fig) significantly increased in OP-treated compared to water-treated mice. Together, the trend towards increased renal GSLs and significant increases in urine GSLs at the endpoint of the study suggest OP treatment increased rather than decreased GSL levels. To determine if OP treatment can increase GSLs, we stimulated primary mesangial cells from pre-disease MRL/lpr mice in the absence or presence of OP. OP significantly decreased NEU activity (Fig 5A) and increased HexCers, LacCers, and GM3s (Fig 5B), as well as Cers, So, and DHSo in stimulated lupus prone mesangial cells (Fig 5C). These *in vitro* results suggest blocking GSL catabolism by inhibiting NEU activity (with OP) may result in accumulation of GSLs in lupus prone mice.

### Inhibiting GlcCer synthase decreased renal GSL levels in MRL/lpr mice

Results from the OP study suggest inhibiting NEU-mediated GSL catabolism may increase rather than decrease already elevated GSL levels, which may exacerbate disease. Therefore, we performed a pilot study to determine if inhibiting the GSL synthesis pathway will reduce GSLs levels. For this study, female MRL/lpr mice were treated with the experimental, proprietary GlcCer synthase inhibitor Genz-667161, which was used to treat mouse models of Gaucher disease and amyotrophic lateral sclerosis [26, 27]. MRL/lpr mice were treated with Genz-667161 at 0, 1, 3, 10, or 20 mg/kg/day. Treatment was started at 12–13 weeks of age for four weeks. Twenty-four-hour urine collections were obtained just prior to treatment and weekly until the end of the study. Serum was collected at the end of the study.

**Fig 6:**
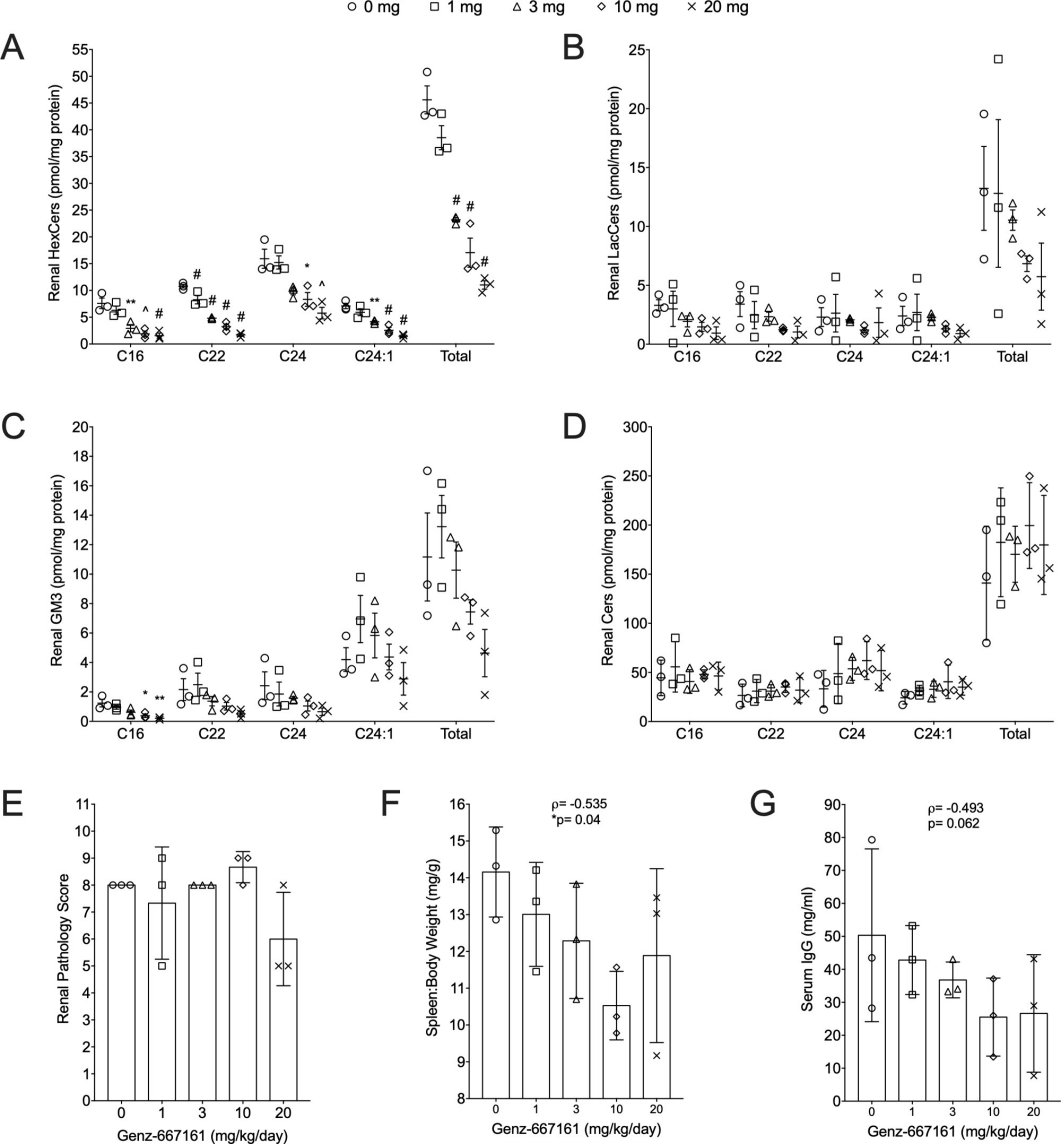
Decreased renal GSLs, splenomegaly, and serum IgG in MRL/lpr mice treated with a GlcCer synthase inhibitor. Female MRL/lpr mice were treated with 0, 1, 3, 10, or 20 mg/kg/day of the GlcCer synthase inhibitor Genz-667161 (n = 3 for each treatment group). Lipids were quantified in renal cortex homogenates at the endpoint of the study. All chain lengths of renal HexCers (A), LacCers (B), GM3s (C), and Cers (D) were quantified. The major chain length species and the total of all chain lengths (Total) are presented for each animal with means and standard errors indicated. There is a significant negative correlation between dose and: HexCer (p<0.001 for all chain lengths); GM3 (p< 0.01 for C16, C22, C24, Total and p<0.05 for C24:1); and LacCer (p<0.05 for C22 and Total). P-values for pairwise comparisons between each treatment group with untreated (0 mg) are presented on the graphs: *p≤0.05, **p≤0.01, ^p≤0.005, #p≤0.001. Renal pathology scores (E), Ratio of spleen weight to body weight (F), and Serum IgG levels (G) were measured at the endpoint of the study. P-values for correlations between dose and outcome in E-G are provided on the graphs.

There was a significant negative correlation between dose with HexCer levels for all chain lengths (p< 0.001 for all) (Fig 6A). Pairwise differences between Genz-667161 doses were also evaluated. Mean differences between each dose and untreated (0 mg) by chain length for each lipid were compared and p-values for the pairwise comparisons are provided on the graphs for each GSL. The most notable pairwise effects were on HexCers levels. All doses ≥3 mg showed significant decreases for all chain lengths of HexCer compared to untreated (0 mg) mice (Fig 6A, p-values indicated on graph). Moreover, the 20 mg dose also resulted in significant decreases for C22 (p = 0.006), C24:1 (p = 0.018), and total (p = 0.016) HexCers relative to the 3 mg dose (Fig 6A). Genz-667161 blocks synthesis of Cers to GlcCers; therefore, the decrease in HexCers is due to decreased GlcCers and not GalCers. There were also significant effects observed on LacCer and GM3 levels. Dose was negatively correlated with C22 and total LacCers levels (p< 0.05) (Fig 6B) and with all GM3 chain lengths at p< 0.01 except C24:1, which was at p< 0.05 (Fig 6C). When pairwise comparisons among doses were evaluated, decreases for each LacCer chain length did not reach significance (Fig 6B) and only C16 GM3 at 10mg and 20mg doses compared to 0 mg reached significance (Fig 6C, p-values indicated on graph). There were no significant differences in Cers C16, C22, C24, C24:1, or total Cer with any dose (Fig 6D).

Based on the significant decreases in renal GSL levels, the effects of Genz-667161 on renal disease and systemic disease were evaluated. The MRL/lpr strain shows significant variability in onset and extent of disease expression with respect to nephritis. Since the three mice in the untreated group all showed low levels of urine albumin, effects of drug treatment on urine albumin was inconclusive (S2 Fig). Renal pathology score (Fig 6E) was not significantly associated with dose of Genz-667161 (treating dose as either continuous- p = 0.112, or categorical- p = 0.219). However, spleen to body weight ratio was negatively correlated (Fig 6F, ρ = -0.535, p = 0.040) and serum IgG levels were marginally correlated (Fig 6G, ρ = -0.493, p = 0.062) with increasing dose. These results suggest inhibiting the GSL synthesis pathway may provide a therapeutic effect in female lupus mice.

## Discussion

Glycosphingolipids are neutral lipids that play important roles in cellular signaling in many cell types. GSLs are enriched in the kidney and GSL metabolism is altered in several kidney diseases, including lupus nephritis [6, 7, 10, 13]. GSL metabolism also was shown to be altered in T cells from patients with lupus [28–31] and in lupus mice [24, 32]. Specifically, we demonstrated that in lupus mice, LacCers, GlcCers, and NEU activity levels were increased in the kidney of mice with worse proteinuria [13, 14] and decreased in T cells of lupus mice with improved disease [24]. Although these results suggest the increased LacCer and GlcCer levels in the kidney were likely a result of increased catabolism of gangliosides due to increased NEU activity, ganglioside levels were not previously analyzed. In this study, we demonstrated that, despite increased NEU activity, renal GM3 levels were actually higher in kidneys of nephritic MRL/lpr mice compared to non-nephritic MRL/MpJ mice. Moreover, we demonstrated SMs, S1P, So, DHS1P, and DHSo are also elevated in the kidneys of nephritic MRL/lpr mice compared to the kidneys of non-nephritic MRL/MpJ mice. Our previous results [13] and the results presented here suggest the upregulation of the GSL pathway in the LN kidney may be due in large part to a general increase in lipids that feed into the GSL pathway through Cer, which we observed were elevated for many more Cers chain lengths in this set of mice in contrast to our previous results [13]. Determining the mechanisms by which the renal GSLs accumulate will likely provide critical insight into the pathophysiology of LN. Since the message level of GM3 synthase was observed to be similar while NEU activity was increased [13], the higher GM3 levels in the MRL/lpr kidney could be due to increased GM3 synthase activity and/or increased catabolism of more complex sialic acid-containing gangliosides to GM3.

Four mammalian NEUs exhibiting different tissue specificities have been identified. NEU1 is the major NEU expressed in the kidney with Neu1 message levels at approximately 10-50-fold higher than Neu3 in human and mouse while Neu2 and Neu4 are expressed at low to undetectable levels in the kidney [14, 33–35]. NEU1 and NEU3 protein expression within the kidney has not been analyzed in detail; however, we demonstrated NEU1 and NEU3 expression in the glomeruli of mouse renal sections appear to be higher in lupus mice with nephritis compared to lupus mice without nephritis [14]. Primary mesangial cells (MCs) from kidneys of MRL/lpr mice upregulated NEU activity and knocking down Neu1 message levels or blocking NEU activity significantly decreased IL-6 secretion upon stimulation *in vitro* [14]. Together, these results suggested accumulation of renal HexCers and LacCers in lupus mice [13, 14] may be due to increased break down of gangliosides by NEUs. Based on these observations we hypothesized that blocking NEU activity may normalize GSLs levels and improve disease. However, our results here blocking NEU activity and the catabolic pathway using the NEU inhibitor OP in MRL/lpr mice resulted in a trend towards increased renal and significantly increased urine GSL levels. Blocking NEU activity with OP in primary lupus serum-stimulated MCs *in vitro* also resulted in a significant increase in GSLs.

As noted above, MRL/lpr kidneys accumulate HexCers, LacCers, and NEU activity increases as disease progresses [13], which suggested increased catabolism may be responsible for the accumulation of GSLs. Our results here demonstrate that GM3 also accumulates during disease despite the increase in NEU activity. If GSL accumulation in MRL/lpr kidneys is due to upregulation of the synthesis pathway, blocking the catabolic pathway could potentially cause *further* accumulation of renal GSLs (see Fig 3D). We speculate the observed increase in renal NEU activity as disease progresses we reported previously [13, 14] may be a response to elevated GSL metabolism and reduce GM3s accumulation in the kidney. Interestingly, a complete knockout of NEU1 in the C57BL/6 strain resulted in pathological effects similar to those observed in lupus, including splenomegaly and proteinuria [36]. Hence, if increased NEU activity is a protective response for removing accumulating GM3s (and other gangliosides) in LN, blocking NEU may actually exacerbate disease. Thus, the trends of increasing renal GSLs in response to treatment with OP may be specific to lupus or specifically the MRL/lpr strain.

It is important to note that there are several potential limitations to our study that also could account for the lack of significant effects on the renal levels of GSLs or disease measures. First, OP was developed as a competitive inhibitor of viral NEU and is not as effective against mammalian NEUs. Several studies demonstrated OP blocks endogenous mammalian NEU activity *in vitro* and *in vivo* with concentrations in the μM range depending on the cell type and study (compared to nM range for viral NEUs) [20, 37–43]. We chose a 10 mg/kg dose based on previous studies in mice demonstrating it was effective in blocking mammalian neuraminidase activity *in vivo* to decrease GM1 levels in T cells [20] and reduce bleomycin-induced lung fibrosis [21]. Although many of these studies suggest OP inhibits NEU1, the major NEU expressed in the kidney, activity of the other NEUs could be compensating if OP does not effectively inhibit all four mammalian NEUs. Second, OP is a prodrug for the active metabolite oseltamivir carboxylate (OC), a structural homolog of sialic acid, and the plasma half-life of OC is approximately 6–10 hours after oral administration. Although a 10 mg/kg/day OP dose in mice was shown to have similar plasma levels of OC as humans administered 75 mg/kg OP twice daily, [19], the once daily administration of OP in this study may not have sustained a steady-state reduction in NEU activity in the kidney, especially since we did not observe any difference in renal NEU activity at the end point of the study. Therefore, the dosage used may have been insufficient to block any, or all, NEU activity. Ideally, development of specific mammalian NEU inhibitors would more accurately determine how, or if, blocking all NEU activity or the activity of a specific NEU will impact renal GSL levels and disease measures in LN.

The unexpected elevated GM3 levels in the MRL/lpr mice compared to the non-nephritic MRL/MpJ parental strain and the results of the OP-treatment study suggest elevated GSL metabolism may be due to continued or increased GSL synthesis rather than increased GSL catabolism. We therefore, tested different doses of a GlcCer synthase inhibitor developed by Genzyme to block the synthesis of GlcCer from Cer. Although this was a pilot study with only three mice per dosage group, the observed significant dose-responsive decrease in GSLs levels including HexCers and GM3s, and negative correlations of splenomegaly and serum IgG with increasing dose suggest strongly that targeting the GSL synthesis pathway may normalize renal GSL levels and improve disease. Another Genzyme GlcCer synthase inhibitor (Genz-123346) reduced renal GlcCer and GM3 levels and improved disease in a mouse model of PKD [7]. Thus, GSL metabolic dysfunction may be a common pathophysiologic pathway in LN and PKD. Based on our pilot study, a larger study with more mice and a longer treatment period is warranted to definitively determine if blocking the GSL synthesis pathway will significantly impact both renal disease and systemic disease.

Studies to identify the mechanisms mediating elevated GSL metabolism during disease progression are needed to better understand the underlying mediators of LN. We previously showed mRNA expression of the synthesis pathway enzymes are not significantly increased in MRL/lpr kidneys [13]. Therefore, upregulation of the synthesis pathway may be a result of increased activity of one or more synthesis enzymes or increased availability of Cers generated from accumulated SMs, So, or DHSo. Alternatively or additionally, infiltrating immune cells may be contributing to the observed elevated renal GSLs and/or NEU activity levels. These potential mechanisms are under investigation and remain to be determined. Defining the mechanisms by which renal GSL metabolism becomes elevated and the pathophysiological effects of elevated GSLs will provide a greater understanding of LN disease development and progression.

In conclusion, we demonstrated renal GM3s are elevated in LN mice despite increased levels of renal NEU activity that catabolizes GM3s. Additionally, we demonstrated SMs and several sphingolipids that feed into the GSL pathway through Cer are also elevated in LN mice. The OP and Genz-667161 treatment study results suggest blocking the GSL catabolic pathway may further elevate renal GSL levels while blocking the synthesis pathway may be effective in normalizing GSL metabolism in nephritic lupus mice. This may be translationally relevant to human patients with lupus nephritis who have elevated urine GSLs levels [13]. It is also possible that blocking both the synthesis and catabolic pathways may be necessary to effectively normalize renal GSL metabolism and attenuate disease expression/progression in lupus nephritis. Alternatively, upregulated GSLs may be playing a protective role, and normalizing renal GSL metabolism may exacerbate nephritis. Hence, further studies to understand the pathophysiologic impact of elevated GSL metabolism in lupus nephritis is needed.

## Supporting information

S1 Fig Urine Cers and SMs and change in urine lipids in OP-treated MRL/lpr mice. HexCers, LacCers, Cers, and SMs were measured in baseline and endpoint 24-hr urine collections and normalized to urine creatinine (UCr). Levels of Cers (A) and SMs (B) in endpoint urine samples. Difference from baseline to endpoint levels of HexCers (C), LacCers (D), Cers (E), and SMs (E). Major chain length species (C16, C22, C24, C24:1) and total of all chain-lengths (Total) of each lipid are presented for each animal and means with standard deviations are provided for all graphs. Water, water-treated MRL/lpr mice; OP, OP-treated MRL/lpr mice.
(TIFF)

S2 Fig Urine albumin levels in Genz-667161-treated mice. Urine albumin was measured in 24 hr urine samples one day prior to beginning treatment and one day prior to euthanasia. The change from Pre-treatment to Endpoint is presented. Means with standard deviations are presented.
(TIFF)
