# Supplementary figures and images for: Targeting glycosphingolipid metabolism as a potential therapeutic approach for treating disease in female MRL/lpr lupus mice
Source: PLoS One. 2020 Mar 18;15(3):e0230499. doi: 10.1371/journal.pone.0230499 (PMC7080257; doi:10.1371/journal.pone.0230499)

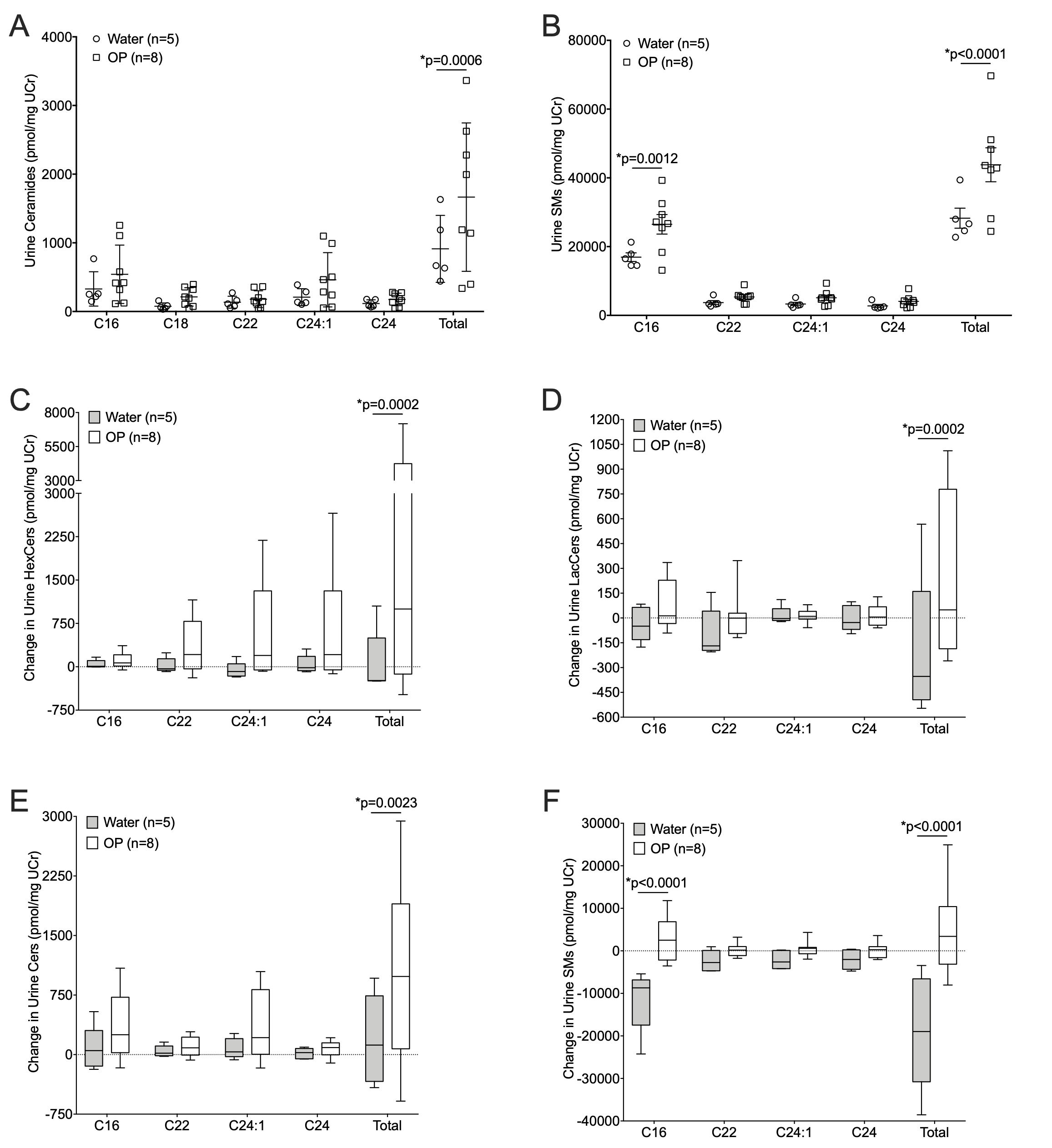

Supplement: S1 Fig — HexCers, LacCers, Cers, and SMs were measured in baseline and endpoint 24-hr urine collections and normalized to urine creatinine (UCr). Levels of Cers (A) and SMs (B) in endpoint urine samples. Difference from baseline to endpoint levels of HexCers (C), LacCers (D), Cers (E), and SMs (E). Major chain length species (C16, C22, C24, C24:1) and total of all chain-lengths (Total) of each lipid are presented for each animal and means with standard deviations are provided for all graphs. Water, water-treated MRL/lpr mice; OP, OP-treated MRL/lpr mice. (TIFF) [file pone.0230499.s001.tiff]

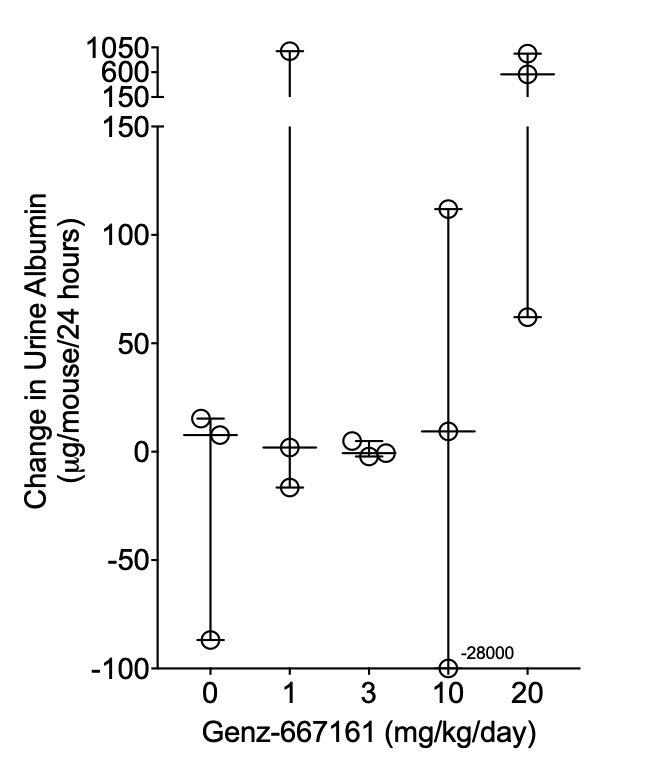

Supplement: S2 Fig — Urine albumin was measured in 24 hr urine samples one day prior to beginning treatment and one day prior to euthanasia. The change from Pre-treatment to Endpoint is presented. Means with standard deviations are presented. (TIFF) [file pone.0230499.s002.tiff]
